# Supplementary material for: Metastability relationship between two- and three-dimensional crystal structures: a case study of the Cu-based compounds
Source: Sci Rep. 2021 Jul 16;11:14588. doi: 10.1038/s41598-021-94034-6 (PMC8285482; doi:10.1038/s41598-021-94034-6)
Supplement: Supplementary file 1 — Supplementary Information. [file 41598_2021_94034_MOESM1_ESM.pdf]

Supplementary Information for "Metastability relationship between two- and three-dimensional crystal structures: A case study of the Cu-based compounds"

Shota Ono

Department of Electrical, Electronic and Computer Engineering, Gifu University, Gifu 501-1193, Japan

Figures S1-S6 show the phonon DOS of CuX in the BHC, B_h, $L1_1$, BSQ, B2, and $L1_0$ structures. The phonon DOS of CuK, CuRb, and CuCs in the B_h and L_1 structures, CuRb and CuCs in the BSQ structure, and CuCs in the $L1_0$ structure were omitted because the optimized structures were not obtained. This might be due to the immiscibility between the Cu and the alkali metals, so that it may be desirable to investigate other structures such as the Cu-rich phase, the alkali metal-rich phase, and/or the surface alloys on another substrate.

CuCr, CuCu, CuAg, and CuAu in the B2 structure have the lowest negative energies of -1.6, -4.2, -3.0, and, -0.5 meV, respectively, which are small compared to their maximum phonon energy. It might be difficult to identify that these are unstable at a glance. In addition to the numerical data of the phonon DOS, we also checked that along the tetragonal Bain path (see Fig. 5) the total energy curve around the B2 structure shows the negative curvature, which determined that CuCu, CuAg, and CuAu are unstable as shown in Fig. 3. CuCr in the B2 structure was dynamically stable and unstable when $3 \times 3 \times 3$ q grid and $4 \times 4 \times 4$ q grid were used, respectively. As listed in Table II in the main text, CuCr in the B2 structure has ferromagnetic phase, while CuCr in the other structures has nonmagnetic phase. The magnetic effect will play an important role in the dynamical stability.

The formation energy and lattice parameters of CuX in the BHC, B_h, $L1_1$, BSQ, B2, and $L1_0$ structures are provided in the file of "E_j_per_cell_eV_and_alat_ang.xlsx". For BHC and BSQ structures, the total thickness is given by 2δ. For some compounds, the $L1_0$ structure is the same as the B2 structure: CuX with X = K, Rb, group 2 (Be, Mg, Ca, Sr, and Ba), group 3 (Sc, Y, and Lu), group 4 (Ti, Zr, and Hf), Pd, Cd, Hg, Tl, and group 14 (Sn and Pb). This is understood from the Fig. 5 in the main text: an almost parabolic curve is observed, yielding $c/a = 1$ in the tetragonal path. Also note that CuCa, CuSr, CuBa, CuSc, CuY, and CuLu have the B1 structure rather than the $L1_1$ structure because the primitive vectors are $a(0, v, v)$, $a(v, 0, v)$, and $a(v, v, 0)$, yielding $\cos\gamma = 0.5$. An almost flat curve around $\cos\gamma = 0.5$ in the trigonal Bain path in Fig. 6, where the unit cell volume of the B2 structure is assumed, may reflect the stability of the B1 structure.

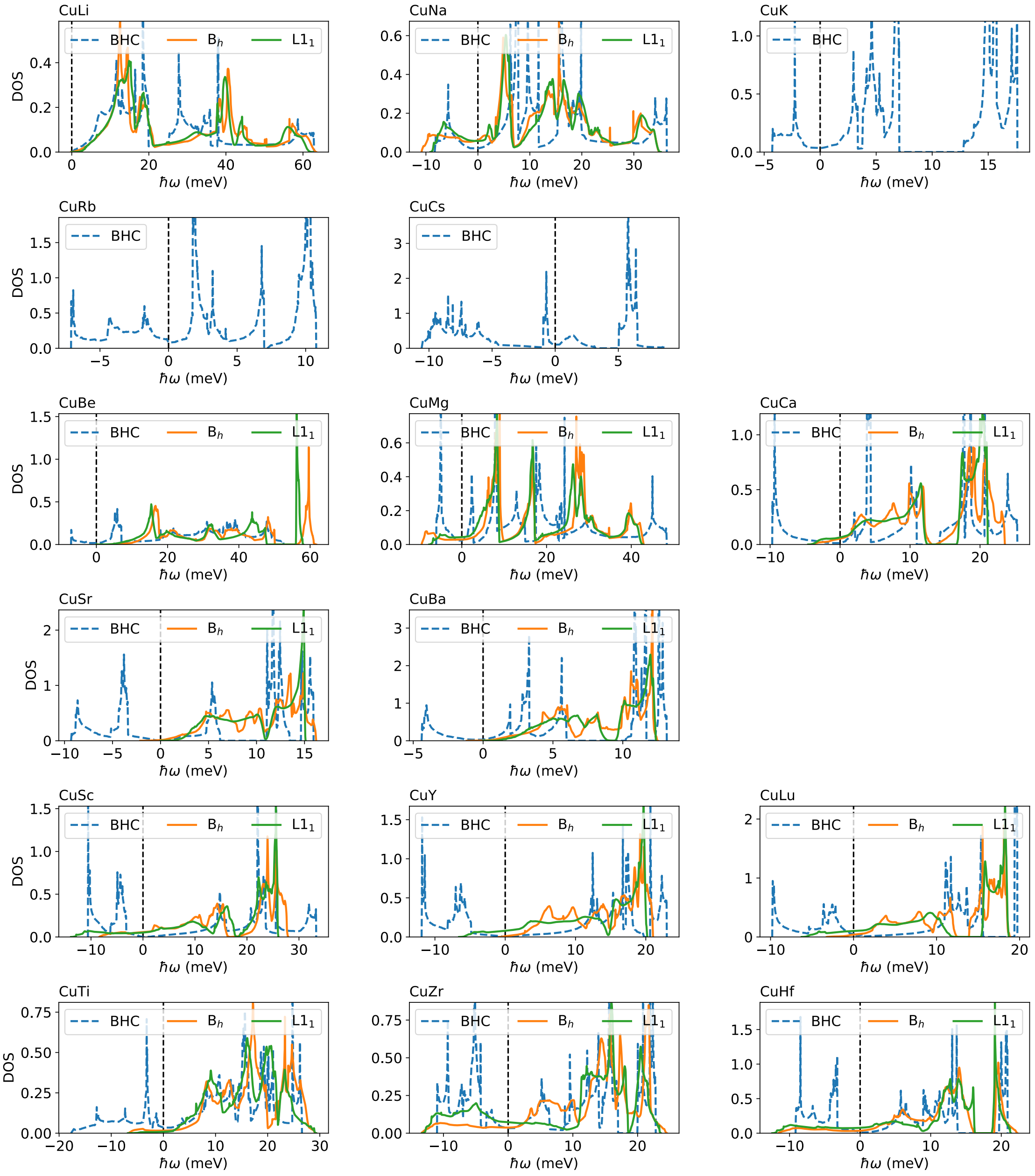

FIG. S1: The phonon DOS of CuX in the BHC, Bh, and L1$_1$ structures for X = group 1, 2, 3, and 4 metals.

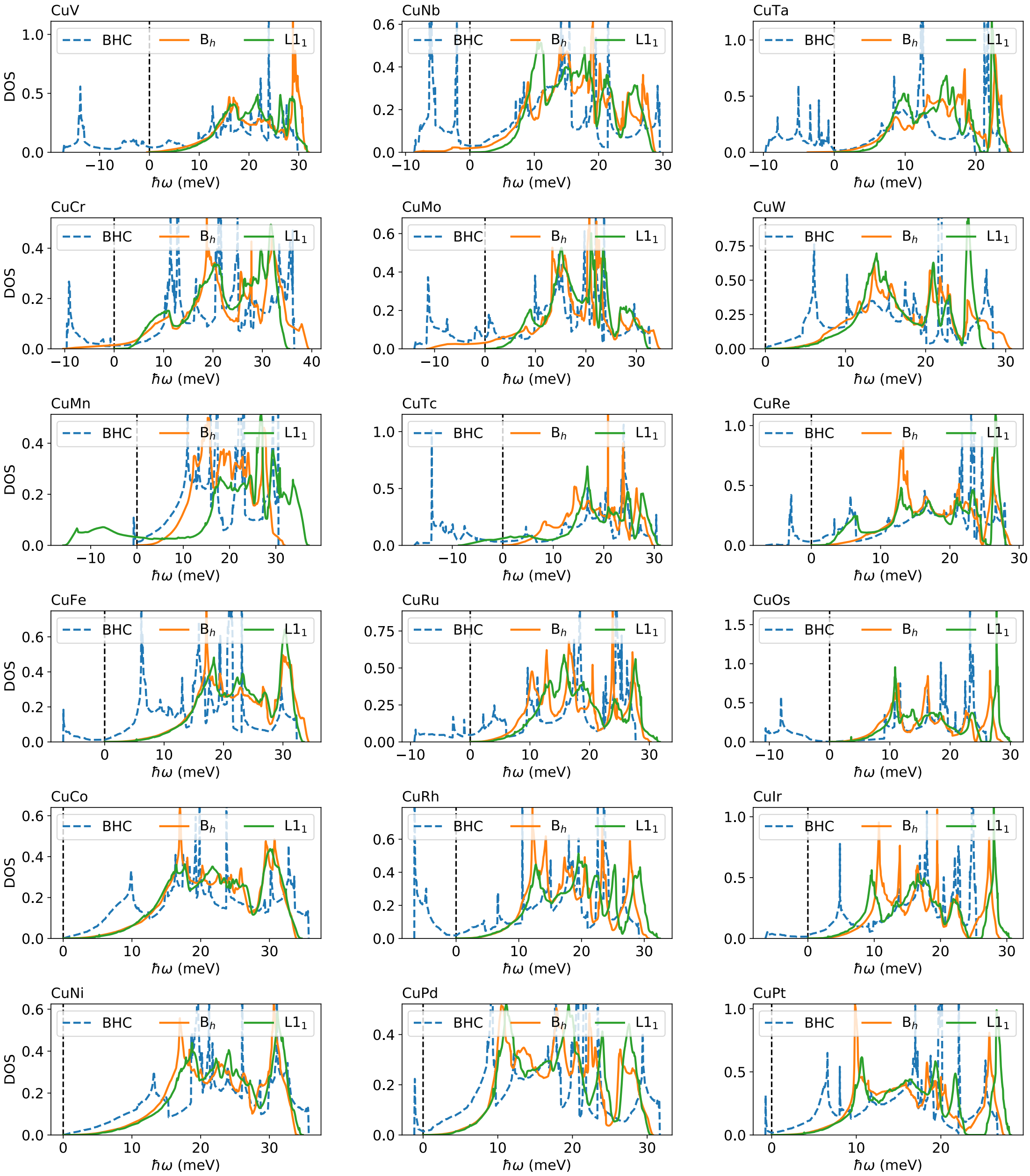

FIG. S2: Same as Fig. S1 but for X = group 5, 6, 7, 8, 9, and 10 metals.

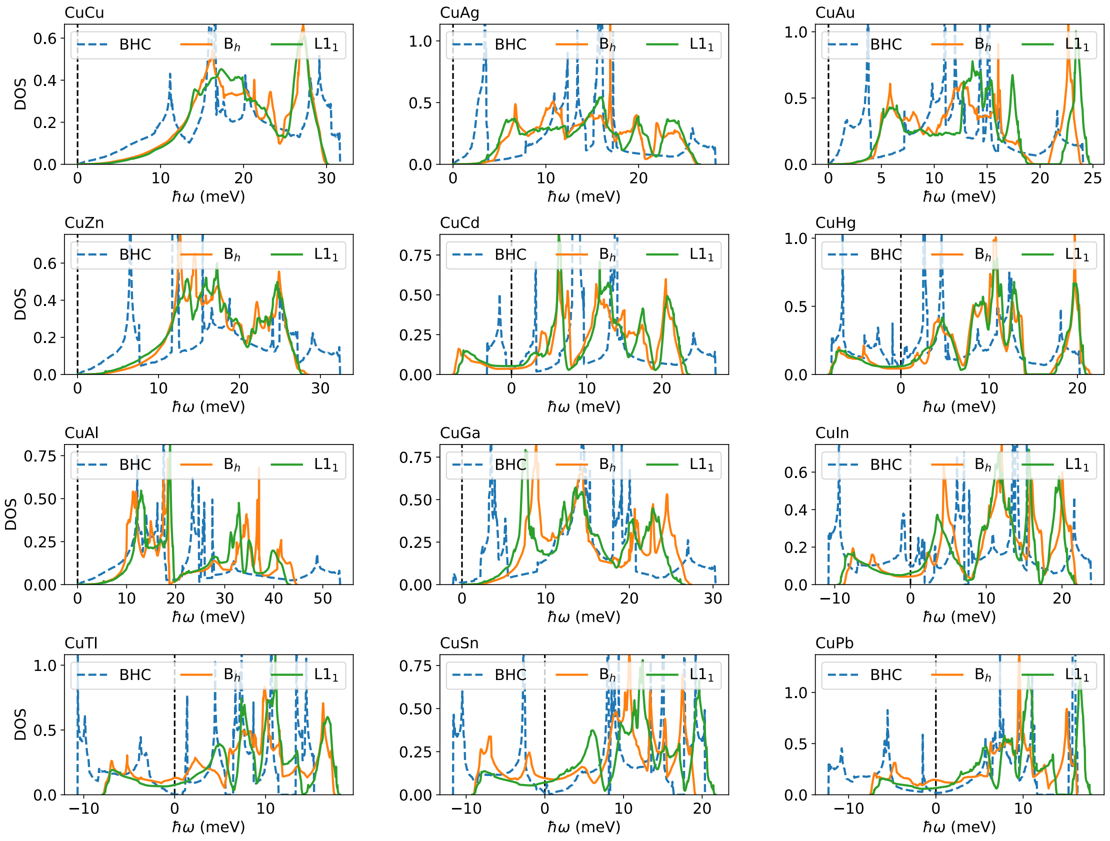

FIG. S3: Same as Fig. S1 but for X = group 11, 12, 13, and 14 metals.

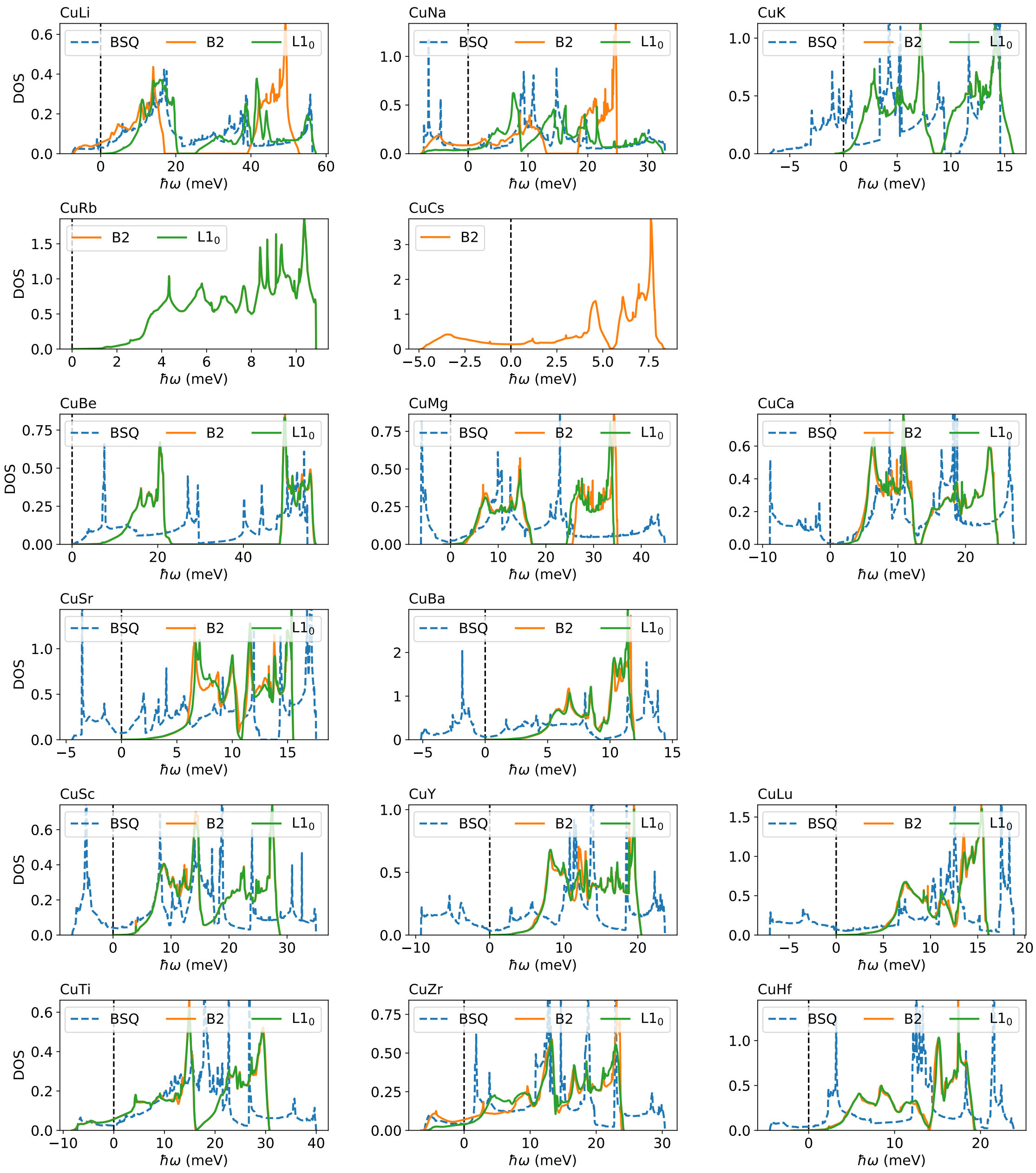

FIG. S4: The phonon DOS of CuX in the BSQ, B2, and L1$_0$ structures for X = group 1, 2, 3, and 4 metals. Except for X = Li, Na, and Cs, the curves of the B2 and L1$_0$ structures almost overlap because the optimized L1$_0$ structure has $c/a \simeq 1$.

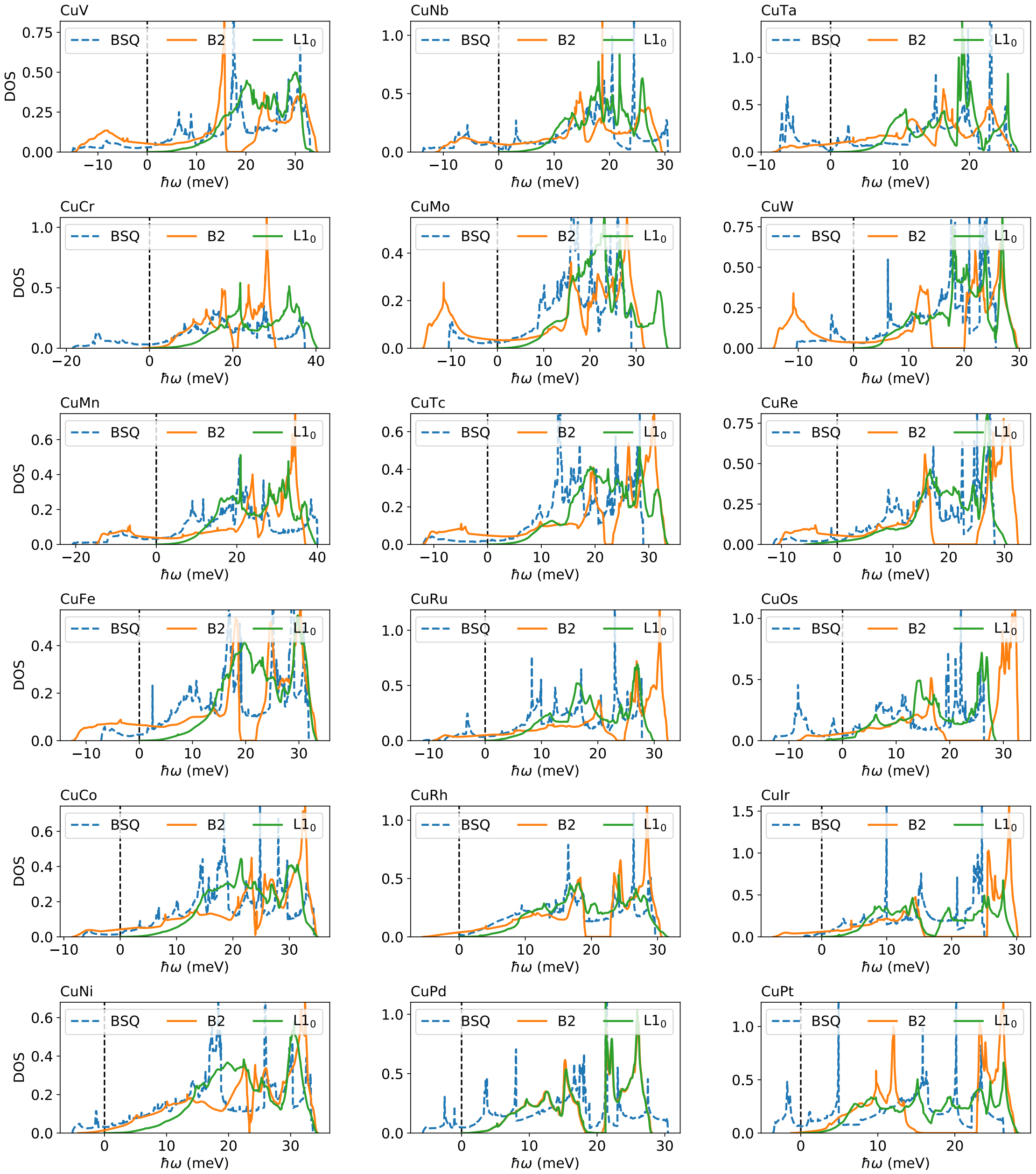

FIG. S5: Same as Fig. S4 but for X = group 5, 6, 7, 8, 9, and 10 metals. The curves of the B2 and L1_0 structures almost overlap for CuPd.

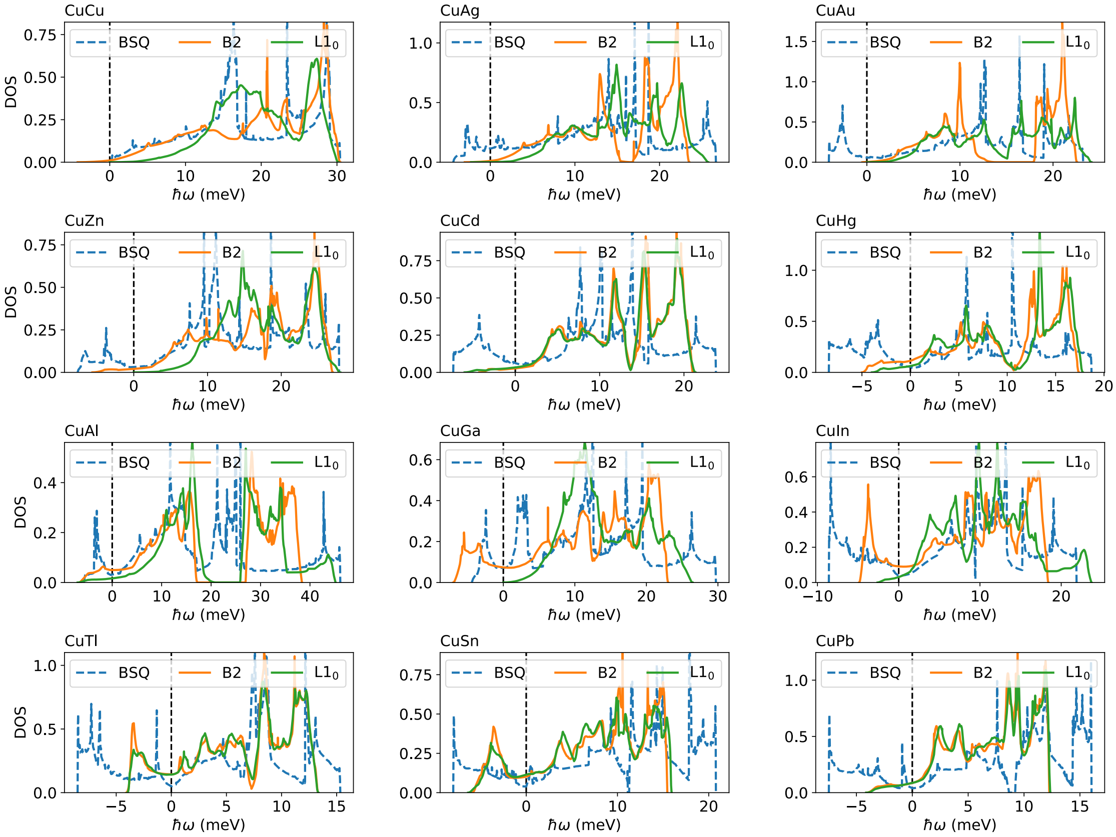

FIG. S6: Same as Fig. S4 but for X = group 11, 12, 13, and 14 metals. The curves of the B2 and L1$_0$ structures almost overlap for CuCd, CuHg, CuTl, CuSn, and CuPb.
